# Assessing the Virologic Impact of Archived Resistance in the Dolutegravir/Lamivudine 2-Drug Regimen HIV-1 Switch Study TANGO through Week 144

**DOI:** 10.3390/v15061350

**Published:** 2023-06-11

**Authors:** Ruolan Wang, Jonathan Wright, Parminder Saggu, Mounir Ait-Khaled, Riya Moodley, Chris M. Parry, Thomas Lutz, Daniel Podzamczer, Richard Moore, Miguel Górgolas Hernández-Mora, Clifford Kinder, Brian Wynne, Jean van Wyk, Mark Underwood

**Affiliations:** 1 ViiV Healthcare, 406 Blackwell Street, Suite 300, Durham, NC 27701, USAmark.r.underwood@viivhealthcare.com (M.U.); 2 GSK, 980 Great West Road, Brentford TW8 9GS, Middlesex, UK; 3 ViiV Healthcare, 980 Great West Road, Brentford TW8 9GS, Middlesex, UK; 4 Infektiologikum, Stresemannallee 3, 60596 Frankfurt am Main, Germany; 5 Hospital Universitari de Bellvitge, Carrer de la Feixa Llarga, s/n, 08907 L’Hospitalet de Llobregat, Barcelona, Spain; 6 Northside Clinic, 370 St Georges Rd, Fitzroy North, VIC 3068, Australia; 7 Jiménez Díaz Foundation University Hospital, Av. de los Reyes Católicos, 2, 28040 Madrid, Madrid, Spain; mgorgolas@fjd.es; 8 AIDS Healthcare Foundation–The Kinder Medical Group, 3661 S Miami Ave Suite 806, Miami, FL 33133, USA

**Keywords:** antiretroviral therapy, 2-drug regimen, HIV-1, resistance, switch, virologic response

## Abstract

The TANGO study (ClinicalTrials.gov, NCT03446573) demonstrated that switching to dolutegravir/lamivudine (DTG/3TC) was non-inferior to continuing tenofovir alafenamide-based regimens (TBR) through week 144. Retrospective baseline proviral DNA genotypes were performed for 734 participants (post-hoc analysis) to assess the impact of archived, pre-existing drug resistance on 144-week virologic outcomes by last on-treatment viral load (VL) and Snapshot. A total of 320 (86%) participants on DTG/3TC and 318 (85%) on TBR had both proviral genotype data and ≥1 on-treatment post-baseline VL results and were defined as the proviral DNA resistance analysis population. Archived International AIDS Society–USA major nucleoside reverse transcriptase inhibitor, non-nucleoside reverse transcriptase inhibitor, protease inhibitor, and integrase strand transfer inhibitor resistance-associated mutations (RAMs) were observed in 42 (7%), 90 (14%), 42 (7%), and 11 (2%) participants, respectively, across both groups; 469 (74%) had no major RAMs at baseline. M184V/I (1%), K65N/R (<1%), and thymidine analogue mutations (2%) were infrequent. Through week 144, >99% of participants on DTG/3TC and 99% on TBR were virologically suppressed (last on-treatment VL <50 copies/mL) regardless of the presence of major RAMs. Results from the sensitivity analysis by Snapshot were consistent with the last available on-treatment VL. In TANGO, archived, pre-existing major RAMs did not impact virologic outcomes through week 144.

## 1. Introduction

HIV-1 treatment guidelines traditionally recommended two nucleoside reverse transcriptase inhibitors (NRTIs) and a third core agent, currently an integrase strand transfer inhibitor (INSTI) or protease inhibitor (PI), which have replaced older drugs such as first-generation non-nucleoside reverse transcriptase inhibitors (NNRTIs) due to the prevalence of transmitted drug resistance and tolerability issues. Large randomized controlled trials have shown the safety and efficacy of the 2-drug regimen (2DR), dolutegravir/lamivudine (DTG/3TC), in treatment-naive participants [1], as well as in stable suppressed participants switching to 2DR [2,3,4]. These studies supported the approval of DTG/3TC for treatment of antiretroviral therapy (ART)-naive people with HIV-1 and those who are stably suppressed with a viral load (VL) of <50 copies/mL and no prior virologic failure or known resistance to DTG or 3TC. The most recent European AIDS Clinical Society, International AIDS Society (IAS)-USA, and Department of Health and Human Services guidelines now recommend the DTG/3TC fixed-dose combination 2DR as a preferred regimen for both ART-naive and ART-experienced virologically suppressed people with HIV-1 [5,6,7].

Where available, drug resistance testing of plasma HIV-1 RNA is the standard of care to identify pre-existing resistance before initiating ART [5,6]. However, for stably suppressed individuals on an antiretroviral regimen requesting or requiring a regimen switch for simplification or to increase tolerability, resistance testing with plasma is not recommended, as VLs are below the threshold of resistance assays. Guidelines state that the use of proviral DNA genotyping may provide complementary information to individuals’ treatment and virologic failure history. For individuals on a stable regimen, an exploratory proviral DNA HIV-1 resistance sequencing approach may be used before treatment switch to assess archived resistance mutations [8], and higher virologic failure rates have been seen with regimens that are less than fully active based on the results of proviral DNA genotyping [9]. However, the clinical use of HIV-1 DNA resistance testing has not been fully defined, and discordance may occur when comparing HIV-1 resistance in plasma RNA with resistance in proviral DNA [10,11,12,13]. 

The TANGO study demonstrated that switching to a 2DR of DTG/3TC was non-inferior to continuing a tenofovir alafenamide-based regimen (TBR) in maintaining viral suppression in ART-experienced adults with HIV-1 through 144 weeks [3]. The historical plasma viral RNA resistance genotype was not required for enrollment in TANGO but was considered for inclusion when available, and participants were excluded if they had historical genotype reports with any IAS-USA major NRTI or INSTI resistance-associated mutations (RAMs) present. Here we describe the results of retrospective HIV-1 proviral DNA genotyping and post-hoc analysis to assess archived, pre-existing drug resistance and investigate its impact on virologic response through 144 weeks in the TANGO study.

## 2. Materials and Methods

### 2.1. Ethics

This study was conducted in accordance with the Declaration of Helsinki and national and institutional standards. Approval was obtained from ethics committees at each investigational site. Written informed consent was obtained from all participants before study initiation.

### 2.2. Study Design

Detailed methodology and study design for the TANGO study (ClinicalTrials.gov, NCT03446573) have been previously published [2] and are briefly described below. Participants were excluded if they had any evidence of IAS-USA major NRTI or INSTI RAMs in any historical genotype assay results, if available; any plasma HIV-1 RNA measurement of ≥50 copies/mL within 6 months of screening; a total of ≥2 measurements of ≥50 copies/mL or any measurement of >200 copies/mL within 6 and 12 months of screening; or a prior regimen switch for virologic failure (HIV-1 RNA ≥400 copies/mL). HIV-1 proviral DNA genotyping was conducted retrospectively with the GenoSure Archive assay (Monogram Biosciences, South San Francisco, CA, USA), which uses next-generation sequencing (NGS) to analyze the HIV-1 polymerase region; a bioinformatic filter is used to remove APOBEC3G/3F-induced G to A hypermutations. Although the NGS platform is able to detect variants as low as 1%, resistance substitutions are reported at a mutation frequency cut-off of ≥10% [14] to minimize over-reporting of APOBEC-mediated hypermutations. Participants’ baseline whole blood samples were used for the GenoSure Archive assay. Virologic outcomes based on IAS-USA major NRTI, NNRTI, PI, and INSTI RAMs [15] were determined by the last available on-treatment of HIV-1 RNA through week 144 in the proviral DNA resistance analysis population (PRAP) to assess on-treatment virologic response. The PRAP was based on the intention-to-treat–exposed (ITT-E) population for whom there were available proviral DNA baseline genotypic data, and at least one post-baseline on-treatment HIV-1 RNA VL result available, and reason for withdrawal was not a protocol deviation. Sensitivity analyses were performed using the US Food and Drug Administration (FDA) Snapshot algorithm at week 144 in the proviral DNA resistance Snapshot Analysis population (PRSAP), which was based on the ITT-E population for all participants with available proviral DNA genotypic data. Confirmed virologic withdrawal (CVW) was defined as HIV-1 RNA of ≥50 copies/mL followed by a second consecutive HIV-1 RNA assessment of ≥200 copies/mL. The list of major RAMs used in these analyses was based on the 2022 IAS-USA update. Pre-specified INSTI substitutions (with major IAS-USA INSTI mutations bolded) are [15]: H51Y, **T66I**/A/K, L68I/V, L74M/I, **E92Q**/V/G, Q95K, T97A, **G118R**, **F121Y**, E138A/K/D/T, **G140**A/C/**R**/S, **Y143C/H/R**/K/S/G/A, P145S, Q146P, **S147G**, **Q148N/H/K/R**, V151I/L/A, S153F/Y, **N155H**/S/T, E157Q, G163R/K, G193E, S230R, and **R263K**.

## 3. Results

Of 919 participants screened for the study, 543 (59%) participants had historical genotypic reports available and submitted for eligibility. Of those with submitted historical genotypes, 9/543 (1.7%) participants were excluded at screening due to pre-existing major NRTI resistance. Among these nine ineligible participants, one had M41L and D67N, two had M41L, and the remaining six each had a single mutation identified as M184I, K65R, K219E, K219Q, D67N, or L210W. A total of 743 participants were randomized and 741 received at least one dose of the study treatments (exposed population). Of those treated, 464 (63%) participants had historical genotypes with 221/369 (60%) in the DTG/3TC and 243/372 (65%) in the TBR group. Retrospective proviral DNA testing was performed on available baseline samples for 734/741 (99%) participants from the exposed population, with 330/366 (90%) in the DTG/3TC group and 324/368 (88%) in the TBR group having genotypic results reported. The GenoSure Archive assay failed to provide a result for 80 of the 734 (11%) samples tested. A further 16 participants, 10 on DTG/3TC and 6 on TBR, failed to meet the criteria for inclusion into PRAP, leaving 320 and 318 participants on DTG/3TC and TBR, respectively.

The overall prevalence of any archived major RAMs across four drug classes was 26% in the PRAP (Table 1). 

Archived NRTI RAMs were observed in 7% of participants and the frequency of M184V/I (n = 7; 1%) and K65N/R (n = 2; <1%) was low, being detected as mutation mixtures with wild-type virus in all cases. Major INSTI RAMs were infrequent, being detected in 2% of participants, all as mutation mixtures with wild type. Pre-specified INSTI substitutions were observed in 26% of participants; the most frequent substitutions were polymorphic G193E, L74I, and V151I. Baseline characteristics (e.g., age, sex, HIV-1 subtype, baseline third agent class, median CD4+ cell count) were similar between participants with or without M184V/I (Table S1). Of the seven with archived M184V/I, four participants were in the DTG/3TC group and had a longer median duration of prior ART compared with the other three in the TBR group (Table S1), whereas a similar duration of prior ART was observed in participants without M184V/I in both treatment groups. 

Through week 144, 319/320 (>99%) participants in the PRAP on DTG/3TC and 314/318 (99%) on TBR were virologically suppressed based on their last on-treatment HIV-1 RNA. Participants with major NRTI, NNRTI, PI, or INSTI RAMs identified by proviral DNA sequencing had a similar high virologic response: 81/81 (100%) on DTG/3TC and 87/88 (99%) on TBR, including four with archived M184V/I on DTG/3TC and three with archived M184V/I, as well as two with archived K65N/R on TBR (Table 1). The four participants, one in the DTG/3TC group and three in the TBR group, who were not suppressed did not have any IAS major RAMs at baseline for all four classes of antiretrovirals or any pre-specified INSTI substitutions (Table 2).

**Figure 1:**
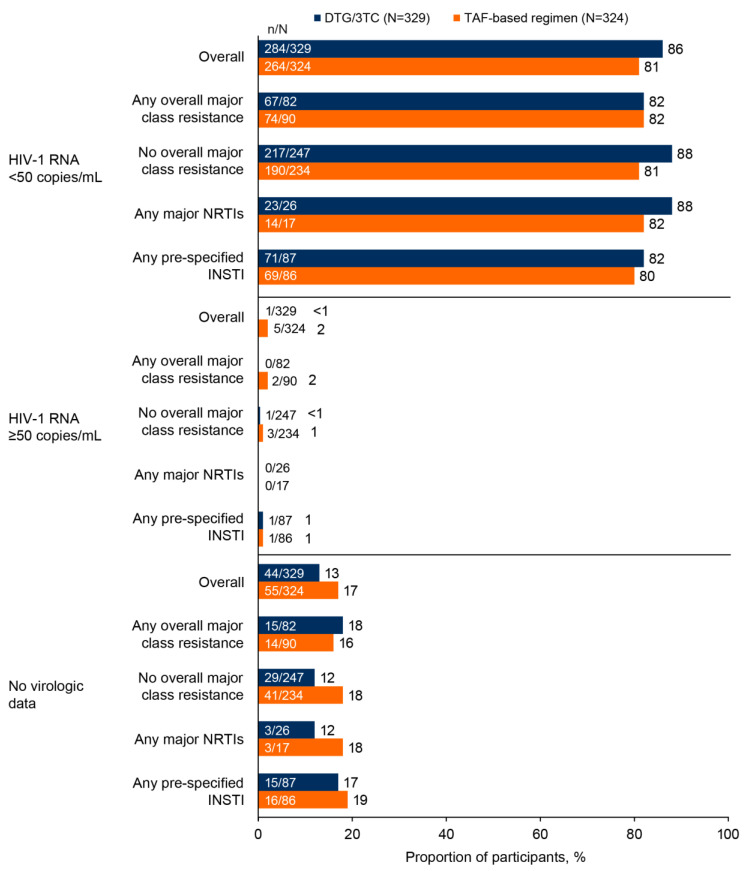
Virologic response (FDA Snapshot algorithm) at week 144 by archived resistance class for all participants in the ITT-E population with available proviral baseline genotypic data (PRSAP) in the DTG/3TC and TAF-based regimen groups. DTG, dolutegravir; FDA, US Food and Drug Administration; INSTI, integrase strand transfer inhibitor; ITT-E, intention-to-treat exposed; NRTI, nucleoside reverse transcriptase inhibitor; PRSAP, proviral DNA resistance Snapshot Analysis population; TAF, tenofovir alafenamide; 3TC, lamivudine.

No participants in the DTG/3TC group met protocol-defined CVW criteria through week 144, while three participants in the TBR group (all without any archived major RAMs) met CVW criteria with no resistance observed at virologic failure time [3]. High suppression rates were also observed at week 144 across both treatment groups, irrespective of the presence of major RAMs, using the FDA Snapshot endpoint in the PRSAP (Figure 1). 

## 4. Discussion

The TANGO study demonstrated the safety and efficacy of DTG/3TC in participants without previous virologic failure or documented resistance [3]. Baseline proviral DNA genotyping showed overall low frequency of archived, pre-existing resistance in line with the population included in the study. Similar results were noted in comparable studies in which a low rate of archived M184V/I was observed [12,16]. A higher rate of archived M184V/I was reported in the pooled analysis of studies GS 1844 and 1878 for participants switching from either PI or DTG to bictegravir/emtricitabine/tenofovir alafenamide (BIC/FTC/TAF). However, in these studies, proviral DNA analysis was performed in a selected sub-population of participants only, such as those with confirmed virologic failure and those who switched to BIC/FTC/TAF with >10 years of prior ART, or with an unknown antiretroviral initiation date [17]. The long or unknown duration of prior ART may reflect the prior use of less effective ART, allowing for resistance development or possibly unrecognized prior treatment failures, which may explain the higher rates of archived resistance in the GS 1844 and 1878 analysis. 

The use of HIV-1 proviral DNA genotyping needs to be better defined and informed with additional clinically based evaluations. Some assessments have shown utility with DNA genotyping: several studies have shown that historical plasma RNA resistance tests were more informative than proviral DNA genotyping for documenting resistance mutations and guiding future treatment regimens in virologically suppressed individuals [10,18], whereas others have found good concordance between historical plasma RNA and proviral DNA genotypes and that resistance detected by proviral DNA genotyping can predict future virologic failure [9]. Another example using proviral DNA genotyping-based ART switch showed no statistically significant change in the probability of HIV-1 RNA of ≥50 copies/mL over time after the switch [19]. Of note, there are a number of confounders of proviral DNA genotyping, including that clinically identified plasma RNA resistance may not always be detected by DNA genotyping [10,12,20]. This may be due to the delayed appearance of resistance in proviral DNA compared with plasma RNA [13] and that detectability of archived DNA genotypic resistance may decrease over time [11]. Other limitations of proviral DNA genotyping include resistance identified in defective (replication incompetent) viruses or mutations at very low levels with no apparent clinical impact. Additionally, the impact of archived mutations in proviral DNA would differ depending on the mutation type and its abundance, as well as the switch regimen components. Furthermore, proper approaches need to be applied when interpreting proviral DNA genotyping reports, as the NGS technology may overestimate APOBEC hypermutation-induced variants depending on reporting thresholds [21,22]. Overall, HIV-1 proviral DNA genotyping results should be used with caution, as reflected by current guideline recommendations [6,23].

In the TANGO study, based on the PRAP, 319 participants (>99%) on DTG/3TC and 314 (99%) on TBR with archived resistance had a VL of <50 copies/mL up to 144 weeks by the last available on-treatment VL. A total of five participants (one on DTG/3TC and four on TBR) had plasma HIV-1 RNA of ≥50 copies/mL by the last available on-treatment VL, with four not having any major baseline archived RAMs and one having pre-existing major NNRTI resistance mutation V108V/I in the TBR group, indicating that, in this study overall, the identification of archived proviral DNA resistance mutations had minimal predictive value on treatment response, as has also been shown in other similar studies [17,24]. It is recognized that the relatively small number of participants with archived resistance and the post-hoc nature of this analysis are limitations on the extent to which these results can be generalized. A strength of this analysis is the robust study design, including randomized treatment assignment and long-term virologic response data, with relatively few withdrawals. In TANGO, virologic suppression was maintained through 144 weeks in participants with archived pre-existing drug resistance treated with DTG/3TC. However, in line with the clinical trial criteria and label, use in individuals with known resistance to either of these drugs should be avoided.

These results, along with other large-scale clinical trials, show that careful consideration of previous treatment history and prior virologic failure, as well as historical resistance reports where available, can help determine whether participants can be successfully switched to DTG/3TC. Further real-world experience in clinical settings using DTG/3TC has also added to the reassuring evidence supporting a successful switch to DTG/3TC in stable virologically suppressed people with HIV-1 [25,26,27,28]. Overall, these results demonstrate no benefit in performing archived DNA resistance testing in a population with no prior virologic failure and no documented prior resistance to INSTIs or NRTIs, as even in the few cases where archived resistance mutations were found, full suppression was maintained through 3 years.

## Figures and Tables

**Table 1:** Prevalence of archived resistance and the most frequent substitutions by drug class at baseline in the proviral resistance analysis population (PRAP) as described in methods.

Baseline Resistance Class, n (%)	DTG/3TC (N = 320)	TAF-Based Regimen (N = 318)	Total (N = 638)
No major RAMs	239 (75)	230 (72)	469 (74)
Any major RAMs	81 (25)	88 (28)	169 (26)
Major NRTI associated ^a^	25 (8)	17 (5)	42 (7)
Any TAM ^b^	9 (3)	5 (2)	14 (2)
A62V	5 (2)	3 (<1)	8 (1)
M184V/I ^c^	4 (1)	3 (<1)	7 (1)
K65N/R ^d^	0	2 (<1)	2 (<1)
Major NNRTI associated ^e^	38 (12)	52 (16)	90 (14)
K103N	12 (4)	17 (5)	29 (5)
E138A	11 (3)	11 (3)	22 (3)
V108I	5 (2)	7 (2)	12 (2)
Major PI associated ^f^	23 (7)	19 (6)	42 (7)
M46I	8 (3)	7 (2)	15 (2)
D30N	5 (2)	2 (<1)	7 (1)
Major INSTI associated ^g^	3 (<1)	8 (3)	11 (2)
G140R	0	3 (<1)	3 (<1)
Q148R	2 (<1)	1 (<1)	3 (<1)
R263K	0	2 (<1)	2 (<1)
Y143H	0	2 (<1)	2 (<1)
Y143C	1 (<1)	0	1 (<1)
Other pre-specified INSTI substitutions ^h^	82 (26)	84 (26)	166 (26)
G193E	32 (10)	29 (9)	61 (10)
L74I	16 (5)	24 (8)	40 (6)
V151I	12 (4)	12 (4)	24 (4)
E157Q	9 (3)	6 (2)	15 (2)
E138D	4 (1)	4 (1)	8 (1)
T97A	5 (2)	3 (<1)	8 (1)
L74M	3 (<1)	4 (1)	7 (1)

DTG, dolutegravir; INSTI, integrase strand transfer inhibitor; NNRTI, non-nucleoside reverse transcriptase inhibitor; NRTI, nucleoside reverse transcriptase inhibitor; PI, protease inhibitor; RAM, resistance-associated mutation; TAF tenofovir alafenamide; TAM, thymidine analogue mutation; 3TC, lamivudine. Note: A participant can have more than one mutation. The numerator is the number of participants with a particular mutation or mutation mixture with wild type detected. ^a^ Other major NRTI RAMs detected <1% in total (n): V75I (6), L74V (3), F77L (1), and K70E (1). ^b^ TAMs including M41L, D67N, K70R, L210W, T215F/Y, and K219E/Q. ^c^ A total of four participants with archived M184V and two with M184I were detected as having mutation mixtures with wild-type virus. ^d^ Participants with archived K65N or R all had mutation mixtures with wild-type virus. ^e^ Other major NNRTI RAMs detected <1% in total: K101E (6), E138K (5), Y181C (4), G190A/S (4), V106A/M (4), Y188C/H/L (4), H221Y (3), E138G (2), M230I/L (2), P225H (2), F227C (1), and K103S (1). ^f^ Other major PI RAMs detected <1% in total (n): V82A (5), V82F/L/S (4), Q58E (4), M46L (3), L90M (2), N88S (2), I47V (1), I50L (1), I84V (1), and N83D (1). ^g^ Participants with archived major INSTI RAMs were all detected as having mutation mixtures with wild-type virus. ^h^ Other pre-specified INSTI substitutions detected <1% in total: T66A (5), G163K/R (5), E138K (2), L68V (2), N155S (2), Q95K (2), G140S (1), and H51Y (1).

**Table 2:** Virologic outcomes by archived resistance category through week 144 using last on-treatment HIV-1 RNA in the proviral resistance analysis population (PRAP ^a^).

Baseline Resistance Class, % (n/N)	Percentage of Participants with Last Available On-Treatment HIV-1 RNA <50 copies/mL	
	DTG/3TC (N = 320)	TAF-Based Regimen (N = 318)
Overall participants	>99 (319/320)	99 (314/318)
Any major RAMs	100 (81/81)	99 (87/88)
No major RAMs	>99 (238/239)	99 (227/230)
Any major NRTI RAMs	100 (25/25)	100 (17/17)
No major NRTI RAMs	>99 (294/295)	99 (297/301)
Any major INSTI RAMs	100 (3/3)	100 (8/8)
No major INSTI RAMs	>99 (316/317)	99 (306/310)
Any pre-specified INSTI substitutions	100 (82/82)	99 (83/84)
No pre-specified INSTI substitutions	>99 (237/238)	99 (231/234)
Any major NNRTI RAMs	100 (38/38)	98 (51/52)
No major NNRTI RAMs	>99 (281/282)	99 (263/266)
Any major PI RAMs	100 (23/23)	100 (19/19)
No major PI RAMs	>99 (296/297)	99 (295/299)

DTG, dolutegravir; INSTI, integrase strand transfer inhibitor; NNRTI, non-nucleoside reverse transcriptase inhibitor; NRTI, nucleoside reverse transcriptase inhibitor; PI, protease inhibitor; PRAP, proviral resistance analysis population; RAM, resistance-associated mutation; TAF tenofovir alafenamide; 3TC, lamivudine. ^a^ PRAP is described in the methods.

## Data Availability

Anonymized individual participant data and study documents can be requested for further research from www.clinicalstudydatarequest.com.

## Supplementary Materials

The following supporting information can be downloaded at: https://www.mdpi.com/article/10.3390/v15061350/s1, Table S1: Baseline Characteristics of Participants With or Without Archived M184V/I in the Proviral Resistance Analysis Population (PRAP ^a^).
