# Supplementary material for: Assessing the Virologic Impact of Archived Resistance in the Dolutegravir/Lamivudine 2-Drug Regimen HIV-1 Switch Study TANGO through Week 144
Source: Viruses. 2023 Jun 11;15(6):1350. doi: 10.3390/v15061350 (PMC10300912; doi:10.3390/v15061350)
Supplement: Supplementary file 1 [file viruses-15-01350-s001.zip › viruses-2421835-supplementary.pdf]

Table S1. Baseline Characteristics of Participants With or Without Archived M184V/I in the Proviral Resistance Analysis Population (PRAP[a])

	**DTG/3TC (N=320)**		**TAF-based regimen (N=318)**	
**Baseline characteristics**	**M184V/I (N=4)**	**Wild-type (N=316)**	**M184V/I (N=3)**	**Wild-type (N=315)**
Age, median (range), y	40.5 (31-61)	40.5 (20-74)	43.0 (28-52)	40.0 (18-73)
Sex, male, n (%)	4 (100)	295 (93)	3 (100)	290 (92)
Race, White, n (%)	4 (100)	253 (80)	2 (67)	242 (77)
HIV-1 subtype, B, n (%)	4 (100)	277 (88)	2 (67)	273 (87)
Region, USA	4 (100)	128 (41)	1 (33)	135 (43)
Baseline third agent class, INSTI, n (%)	3 (75)	249 (79)	3 (100)	250 (79)
Baseline CD4+ count, median (range), cells/mm^3	750.0 (508-1062)	677.5 (133-1671)	575.0 (416-1075)	725 (119-1810)
Duration of ART before Day 1, median (range), mo	72.5 (50.2-158.3)	34.4 (7.3-201.2)	27.7 (9.3-30.3)	36.4 (7.0-147.4)

ART, antiretroviral therapy; DTG, dolutegravir; INSTI, integrase strand transfer inhibitor; RAM, resistance-associated mutation; TAF tenofovir alafenamide; 3TC, lamivudine.
[a]PRAP is described in the Methods.
